# 1.A. Workshop: EU nutrition policies: from rhetoric to practice

**DOI:** 10.1093/eurpub/ckac129.001

**Published:** 2022-10-25

**Authors:** Organised by: EUPHA-LAW, EUPHA-FN, University of Essex (UK), University of Liverpool Law & NCD Unit (UK), European Public Health Alliance, Chair persons: Giuseppe Grosso (EUPHA-FN)

## Abstract

Unhealthy diets result in over a million deaths each year in Europe, and over half the EU adult population is overweight or obese. Since it took office, the von der Leyen Commission published major policy documents which, amongst others, seek to tackle poor nutrition. The 2020 Farm to Fork Strategy claims that “European food is already a global standard for food that is safe, plentiful, nutritious and of high quality”. However, it also acknowledges that the European diet is typically high in fat, sugar, salt and calories and red and processed meat, but low in fruit, vegetables, wholegrains, legumes and nuts. The Strategy notes that the transition to a healthy, sustainable food system - and the concomitant health, economic, societal and environmental benefits this will bring - requires that the food environment should make healthy food easier to choose. Similarly, the 2022 Europe's Beating Cancer Plan accepts that about 40% of cancers in the EU are preventable and, prevention being more effective than any cure, places great emphasis on the promotion of healthier consumption. This workshop will consider two policy interventions that the WHO has recognised as key to the promotion of healthier food environments and that have been at the heart of the EU's strategic documents on nutrition and obesity prevention for now well over 15 years: food labelling and food marketing. This workshop will seek to contextualise the Commission's current proposals on food labelling, and its lack of robust proposals on food marketing, with a view to discussing the extent to which its actions are aligned with its rhetoric. It will also discuss how the Commission can move in the right direction to bring real improvements in these fields in practice. EUPHA-LAW has been supporting the development of effective EU policies on food labelling and food marketing through a number of initiatives. In relation to food labelling, EUPHA-LAW responded to three Commission consultations, members of the Steering Committee held a meeting with the Commissioner's team on the new proposals, co-hosted a major conference on regulating FoPNL with almost 300 attendees from over 39 countries, and participated in workshops specifically intended to inform the impact assessment accompanying the forthcoming proposals. In relation to food marketing, EUPHA-LAW is closely involved in the Food Marketing Initiative supported by over 20 pan-EU federations of public health, consumer and children's rights organisations. This Initiative has called on the EU to adopt a directive imposing EU-wide legally binding restrictions on the cross-border marketing of unhealthy food to children. This would allow the EU to go further than it has to date and therefore align the EU regulatory framework with international recommendations and adopt a children's rights-based approach to such marketing.

**Key messages:**

• The Commission has recognised that front-of-pack nutrition labelling and food marketing are two necessary policy interventions to promote healthier food environments.

• However, the EU’s actions in this field highlight a significant gap between rhetoric and practice which needs to be addressed through the adoption of rights-based approaches.

